# Individualized prediction of risk of ascending aortic syndromes

**DOI:** 10.1371/journal.pone.0270585

**Published:** 2022-06-27

**Authors:** Qais Waleed Saleh, Axel Cosmus Pyndt Diederichsen, Jes Sanddal Lindholt

**Affiliations:** 1 Department of Thoracic-, Cardiac- and Vascular Surgery, Odense University Hospital, Odense, Denmark; 2 Centre for Individualized Medicine in Arterial Diseases (CIMA), Odense, Denmark; 3 Centre of Clinical Excellence in Southern Denmark (CAVAC), Odense, Denmark; 4 Department of Cardiology, Odense University Hospital, Odense, Denmark; Universitaria di Bologna, ITALY

## Abstract

**Objectives:**

Although ascending aortic diameter changes acutely after dissection, recommendation for prophylactic surgery of thoracic aortic aneurysms rely on data from dissected aortas. In this case-control study we aim to identify risk markers for acute and chronic aortic syndromes of the ascending aorta (ACAS-AA). Furthermore, to develop a predictive model for ACAS-AA.

**Methods:**

We collected data of 188 cases of ACAS-AA and 376 controls standardized to age- and sex of the background population. Medical history and CT-derived aortic morphology were collected. For the dependent outcome ACAS-AA, potential independent risk factors were identified by univariate logistic regression and confirmed in multivariate logistic regression. As post-dissection tubular ascending aortic diameter is prone to expand, this factor was not included in the first model. The individual calculated adjusted odds ratios were then used in ROC-curve analysis to evaluate the diagnostic accuracy of the model. To test the influence of post-ACAS-AA tubular ascending aortic diameter, this was added to the model.

**Results:**

The following risk factors were identified as independent risk factors for ACAS-AA in multivariate analysis: bicuspid aortic valve (OR 20.41, p = 0.03), renal insufficiency (OR 2.9, p<0.01), infrarenal abdominal aortic diameter (OR 1.08, p<0.01), left common carotid artery diameter (OR 1.40, p<0.01) and aortic width (OR 1.07, p<0.01). Area under the curve was 0.88 (p<0.01). Adding post-ACAS-AA tubular ascending aortic diameter to the model, negated the association of bicuspid aortic valve, renal insufficiency, and left common carotid artery diameter. Area under the curve changed to 0.98 (p<0.01).

**Conclusions:**

A high performing predictive model for ACAS-AA, free of ascending aortic diameter, can be achieved. Furthermore, we have identified abdominal aortic ectasia as an independent risk factor of ACAS-AA. Integration of potential biomarkers and morphologic variables, derived from undissected aortas, would probably improve the model.

## Introduction

Acute and chronic aortic syndromes of the ascending aorta (ACAS-AA) namely: acute or chronic dissection, rupture, intramural hematoma or penetrating aortic ulcers, are life threatening and require immediate surgery. The prognosis of ACAS-AA is poor as up to 22% of patients die acutely before reaching a hospital [1], and overall in-hospital mortality is 30% [2].

To prevent ACAS-AA, prophylactic surgery is recommended when ascending aortic diameter exceeds 55 mm, with slight modifications if bicuspid aortic valve (BAV) and/or genetic diseases are present [3]. However, recent studies show that most ACAS-AA occur at diameters < 55 mm [2, 4], and some patients do not suffer ACAS-AA regardless of aortic dilatation [2].

The cut-off value of 55 mm is based on data from already dissected aortas. This value has recently been questioned, because ascending aortic diameter expands acutely following dissection [4–7]. However, ascending aortic diameter remains a major indicator for preventive repair, as the relative risk of dissection compared to the background population increases sharply at diameters larger than 45 mm [8]. Consequently, there is a need to explore additional risk factors that can further differentiate the risk of dissection.

Aortic elongation [9] and bovine aortic arch [10] seem to be risk factors for ACAS-AA. Ratios relating aortic diameter to age, body weight and height have been developed, but have not been implemented [11]. The association of supra-aortic vessel diameters have received less attention, as has the presence of abdominal aortic aneurysms or established risk factors for abdominal aortic rupture.

Moreover, diameters of vertebral corporate depend on genetically defined body size. Ratios comparing vertebral corporal diameters to aortic diameter can be easily obtained and might prove useful clinically.

In this paper, we aim to identify risk factors of ACAS-AA, and to develop a predictive multivariate logistic regression model based on computer tomography (CT)-derived aortic variables and medical history.

## Materials and methods

### Setting

We obtained medical records and CT-scans of patients treated at Odense University Hospital, in the department of Cardiac-, Thoracic- and Vascular Surgery and the department of Cardiology, from 01.01.2009 to 01.03.2018. Patients were identified via the patient administrative system, through their social security number. Data was collected retrospectively.

### Participants

#### Cases

We included patients diagnosed with: Acute Stanford type A aortic dissection–symptoms not exceeding 14 days; Chronic Stanford type A aortic dissection–symptoms exceeding 14 days; contained ascending aortic rupture–dissection through all aortic layers except the adventitia; ascending aortic rupture–rupture through all aortic layers; ascending aortic intra mural hematoma–hematoma without a visible intimal dissection flap or inlet; ascending aortic penetrating aortic ulcers–ulceration into the aortic wall in the basis of an atherosclerotic plaque. We excluded iatrogenic- and traumatic dissections, patients who had not undergone CT-scanning before surgery or death, and patients whose CT-scans or medical history were unobtainable.

We verified the diagnoses through surgical- or autopsy reports if available, otherwise we used radiological reports. If none of the above was available, CT-scans were evaluated according to radiologic features described in the literature [12].

#### Controls

For each case, two controls were included. These were comprised of patients who had undergone CT-scanning of the thorax, and preferably of the abdomen as well, for reasons other than ACAS-AA. To mirror the age distribution of the background population [13], controls were included in age groups with the same distribution. The age-groups begin at 35, and end at older than 85, with five-year intervals. The ratio of men to women was set to 1:1. Finally, to limit possible confounders such as surgery or medication, we collected data from index contacts.

### Variables

The presence of ACAS-AA in the ascending aorta was the dependent variable. Suspected independent variables are summarized in Tables 1 and 2.

**Table 1:** Descriptive statistics of independent variables gathered through medical record review, and results of univariate logistic regression analysis, where the presence of acute or chronic aortic syndrome of the ascending aorta is the dependent variable. Continuous variables are presented with mean ± standard deviation, categorical variables with numbers and frequency. “- “= no results due to no/low observations.

Independent variable	Cases	Controls	Univariate analysis results, unadjusted odds ratio (90% Confidence Interval)	*p-*value
**Sex, N (%)**				
Men	113 (60.1%)	188 (50%)	1.04 (1.03–1.05)	< 0.01
Women	75 (39.9%)	188 (50%)		
**Age (years)**	66.33 ± 13.57	58.13 ± 14.12	1.51 (1.11–2.02)	0.02
**Height (cm)**	174.14 ± 10.35	171.79 ± 9.72	1.02 (1.01–1.04)	0.01
**Weight (kg)**	80.00 ± 19.26	75.42 ± 16.87	1.01 (1.01–1.02)	< 0.01
**BMI (kg/m^2^)**	26.21 ± 4.99	25.52 ± 4.95	1.02 (0.99–1.05)	0.12
**BSA–Dubois formula (m^2^)**	1.94 ± 0.25	1.87 ± 0.22	3.33 (1.76–6.31)	< 0.01
**BSA–Mosteller’s formula (m^2^)**	1.95 ± 0.27	1.88 ± 0.24	3.00 (1.65–5.46)	< 0.01
**Family history of acute or chronic syndrome of the ascending aorta, N (%)**	5 (2.7%)	0	1	-
**History of, N (%)**				
Predisposing genetic syndrome	2 (1.1%)	0	1	-
Abdominal/Descending aortic dissection or rupture	9 (4.7%)	4 (1.0%)	4.67 (1.72–12.7)	0.01
Aortic Coarctation	1 (0.5%)	0	1	-
Bicuspid aortic valve	10 (5.3%)	3 (0.7%)	6.98 (2.34–20.83)	< 0.01
Replaced aortic valve	11 (5.8%)	3 (0.8%)	7.72 (2.61–22.79)	< 0.01
COPD	20 (10.7%)	49 (13.0%)	0.79 (0.5–1.27)	0.42
Polycystic kidney disease	0	3 (0.8%)	1	-
Hypertension	86 (46%)	132 (35.1%)	1.58 (1.17–2.14)	0.01
Diabetes	9 (4.8%)	31 (8.2%)	0.56 (0.29–1.06)	0.14
Peripheral arterial disease	3 (1.6%)	12 (3.2%)	0.49 (0.16–1.44)	0.28
Ischemic heart disease	19 (10.2%)	24 (6.4%)	1.65 (0.97–2.81)	0.11
Cerebrovascular disease	25 (13.3%)	21 (5.5%)	2.60 (1.56–4.34)	< 0.01
Renal insufficiency	70 (37.8%)	44 (11.9%)	4.46 (3.11–6.42)	< 0.01
Chronic immunosuppression	15 (8.0%)	18 (4.8%)	1.73 (0.95–3.14)	0.12
Autoimmune diseases	12 (6.4%)	12 (3.2%)	2.08 (1.04–4.14)	0.08
Pheochromocytoma	0	0	1	-
SIRS within 3 months	15 (8.0%)	46 (12.2%)	0.62 (0.37–1.04)	0.13
Pregnancy	0	0	1	-
Smoking				
**Current**	61 (35.7%)	177 (47.4%)	0.33 (0.22–0.47)	< 0.01
**Former**	40 (23.4%)	129 (34.6%)	0.29 (0.19–0.47)	< 0.01
**Never**	70 (37.2%)	67 (17.8%)	-	-
**Medication and Drugs, N (%)**				
Statins	43 (23.1%)	71 (18.9%)	1.29 (0.90–1.84)	0.24
Warfarin/NOAC	21 (11.2%)	21 (5.5%)	2.15 (1.26–3.65)	0.01
Oral glucocorticoids	9 (4.8%)	16 (4.3%)	1.14 (0.56–2.31)	0.75
Bronchodilators	20 (10.7%)	55 (14.6%)	0.70 (0.44–1.11)	0.20
Platelet inhibitors	51 (27.0%)	64 (17%)	1.84 (1.29–2.61)	< 0.01
NSAID	6 (3.2%)	16 (4.3%)	0.75 (0.33–1.67)	0.55
Cocaine	0	0	1	-
Amphetamine	0	1 (0.3%)	1	-
**Blood pressure (mmHg)**				
Systolic	120 ± 32	138 ± 22	0.97 (0.96–0.98)	< 0.01
Diastolic	66 ± 20	81 ± 12	0.94 (0.93–0.95)	< 0.01
Mean arterial pressure	84 ± 22	100 ± 14	0.95 (0.94–0.96)	< 0.01
Pulse pressure	52 ± 23	57 ± 17	0.98 (0.98–0.99)	0.01
**Laboratory values**				
Hemoglobin (mmol/L)	8 ± 1.14	8 ± 1.01	0.67 (0.58–0.77)	< 0.01
Thrombocytes (10^9^/L)	205 ± 71.47	296 ± 108	0.98 (0.98–0.99)	< 0.01
Creatinine (μmol/L)	98 ± 46.38	83 ± 88.53	1.01 (1.00–1.01)	0.09
eGFR (ml/min/1,73 m^2^)	69 ± 23.07	86 ± 21.15	0.96 (0.96–0.97)	< 0.01

**Table 2:** Descriptive statistics of independent variables, gathered through analysis of computer tomography scans, and results of univariate logistic regression analysis where the presence of acute or chronic aortic syndrome of the ascending aorta is the dependent variable. Continuous variables are presented with mean ± standard deviation, categorical variables with numbers and frequency. “- “= no results due to no/low observations.

Independent variable	Cases	Controls	Univariate analysis results, unadjusted odds ratio (90% Confidence Interval)	p-value
**Variations of aortic anatomy, N (%)**				
Bovine arch	35 (18.6%)	53 (14.1%)	1.39 (0.94–2.06)	0.16
Isolated vertebral artery	14 (7.4%)	21 (5.6%)	1.36 (0.75–2.44)	0.38
Aberrant right subclavian artery	0	1 (0.3%)	1	-
Right aortic arch	0	0	1	-
**Arterial vessel diameters (mm)**				
Sinutubular junction	49.31 ± 10.55	31.64 ± 4.55	1.46 (1.38–1.55)	< 0.01
Tubular Ascending Aorta	53.44 ± 10.16	34.92 ± 4.75	1.49 (1.40–1.58)	< 0.01
Ratio to medio-lateral abdominal aortic diameter	0.49 ± 0.23	0.57 ± 0.15	0.01 (0.01–0.03)	< 0.01
Ratio to First Vertebral Corpus	29.5 ± 0.44	19.9 ± 0.15	1.82 (1.66–1.99)	< 0.01
Distal Ascending Aorta	41.78 ± 6.74	31.90 ± 3.89	1.54 (1.45–1.64)	< 0.01
Aortic Arch	31.89 ± 4.60	26.59 ± 3.31	1.44 (1.36–1.52)	< 0.01
Proximal Descending Aorta	29.54 ± 4.35	24.02 ± 3.08	1.54 (1.44–1.64)	< 0.01
Descending Aorta	31.85 ± 5.45	25.37 ± 3.29	1.49 (1.40–1.58)	< 0.01
Proximal Abdominal Aorta	28.18 ± 4.26	23.45 ± 3.23	1.43 (1.35–1.51)	< 0.01
Distal Abdominal Aorta	23.16 ± 4.59	19.14 ± 3.05	1.38 (1.30–1.46)	< 0.01
Infrarenal abdominal Aortic Diameter				
Anterior-posterior	25.14 ± 8.35	19.83 ± 5.59	1.17 (1.12–1.22)	< 0.01
Medio-lateral	25.73 ± 9.74	19.94 ± 5.80	1.16 (1.12–1.20)	< 0.01
Ratio to Third Lumbar Corpus	7.40 ± 2.20	6.00 ± 1.58	1.72 (1.51–1.96)	< 0.01
Brachiocephalic trunk	15.87 ± 2.99	12.54 ± 2.27	1.62 (1.50–1.74)	< 0.01
Left Common Carotid Artery	9.64 ± 2.35	8.10 ± 1.36	1.63 (1.48–1.79)	< 0.01
Left Subclavian Artery	12.67 ± 2.87	11.11 ± 2.16	1.28 (1.21–1.37)	< 0.01
**Other (mm)**				
Aortic length	224.15 ± 26.91	185.56 ± 30.61	1.04 (1.03–1.05)	< 0.01
Aortic width	98.04 ± 15.93	78.37 ± 13.39	1.09 (1.07–1.11)	< 0.01
Aortic height	80.56 ± 12.69	68.63 ± 13.1	1.07 (1.05–1.08)	< 0.01
Aortic Tortuosity	2.33 ± 0.43	2.39 ± 0.38	0.67 (0.45–0.98)	0.08
Diameter of First Thoracic Vertebral Corpus	18.23 ± 1.98	17.58 ± 1.81	1.20 (1.11–1.30)	< 0.01
Diameter of Third Lumbar Vertebral Corpus	33.50 ± 3.45	33.20 ± 3.30	1.02 (0.98–1.07)	0.33
Estimated ascending aortic diameter	34.79 ± 1.30	33.68 ± 1.37	1.57 (1.4–1.77)	< 0.01

#### Medical record review

We gathered data describing weight, height, age, gender, comorbidities, medications, history of illicit drug use, cardiovascular risk factors, family history of aortic disease, predisposing genetic syndromes, laboratory values and blood pressure. Laboratory values and blood pressure were detected at the time of hospitalization for cases, and at the time of index CT-scanning for controls.

Predisposing genetic syndromes included Marfan syndrome, Ehlers-Danlos syndrome type IV, Loeys-Dietz syndrome, and Turner syndrome. Patients with a history of congenital immunosuppression or those with leucopenia (leucocyte levels < 4 x 10^9^ cells/L) and a history of recurrent infections were regarded as chronically immunosuppressed. Autoimmune diseases encompassed Takayasu arteritis, giant cell arteritis, anchylosing spondylitis, morbus Behçet, rheumatoid arthritis, systemic lupus erythematosus, inflammatory bowel disease, Sjögren’s syndrome, Scleroderma, and Hashimoto’s thyroiditis. SIRS was defined according to criteria published in an article by Bone et al. [14].

Active -, previous—and never smokers were defined according to self-reporting. Renal insufficiency was defined as eGFR < 60 ml/min/1,73 m^2^. Those treated with an antihypertensive were regarded as having a history of hypertension. Patients treated with antidiabetics or those who had an HBA1c > 48 were regarded as diabetics. Patients with mechanic aortic valves were separated from patients treated with anticoagulant agents for other reasons. Similarly, patients treated with thrombocyte inhibitors due to ischemic stroke were separated from those treated with the same agents for other reasons.

#### CT evaluation

Values were obtained using Siemens syngo.via® image analysis platform. (Siemens Healthcare A/S, Erlangen, Germany).

CT-derived variables were measured in the same manner as a parallel study, performed in the same setting and on the same sample population, the results of which have already been published [6]. In short, the inner-to-inner aortic diameter was measured perpendicular to the vessel axis from the sinotubular junction to the proximal abdominal aorta. The diameters of the supra-aortic vessels were measured as well.

**Fig 1:**
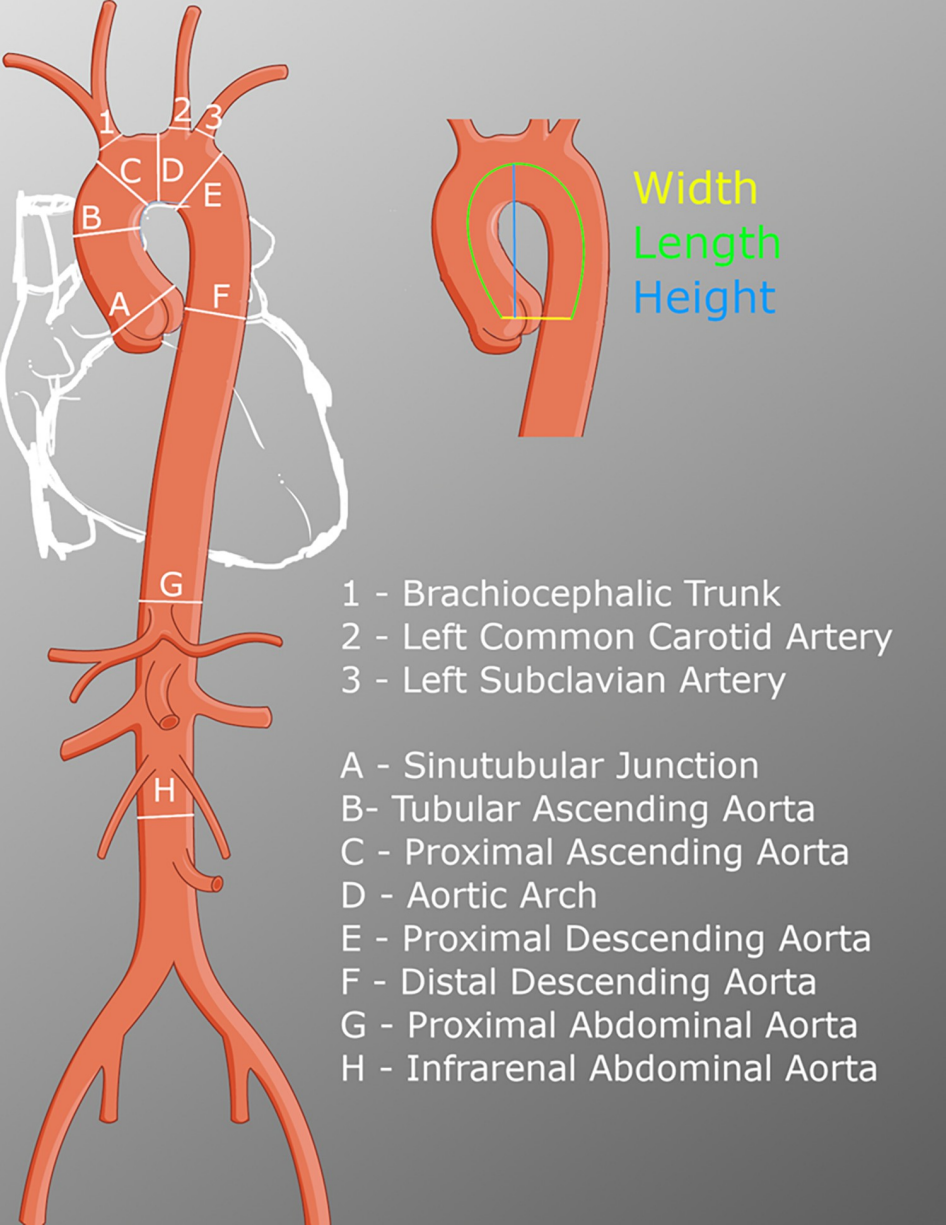
Illustrates the level at which aortic and other vessel diameter and morphology was measured. This figure was created using adapted material from Smart Servier Medical Art, smart.servier.com.

In addition, we measured; the largest infrarenal abdominal aortic diameter in both the mediolateral and anterior-posterior directions; Aortic length–the distance spanning from the sinotubular junction to the corresponding point of the descending aorta in the transverse plane; Aortic width—the direct distance between the sinotubular junction and the corresponding point of the descending aorta in the transverse plane; Aortic height–the distance between the most superior point of the aortic centerline to the transverse level at which the aortic width was measured, in a 90 degree angle; Anterior to posterior diameters of the first thoracic and third lumbar vertebral corporal bodies, perpendicular to the vertebral corporal axis. Finally, anatomic supra-aortic vessel variation was noted. Locations of aortic measurements are illustrated in Fig 1.

#### Calculated data

Body surface area (BSA) was calculated using Mosteller’s and Duboi’s formulae. Tortuosity was defined as the ratio between aortic length and width. We calculated the ratio of the anterior-posterior infrarenal abdominal aortic diameter relative to the diameter of the third lumbar vertebral corpus multiplied by 10 to make the results interpretable. Finally a calculated estimate of ascending aortic diameter derived from a study by Obel et al. [15], was calculated. This estimate relies on age, BSA and sex.

### Statistical analysis

We used STATA 16.1® (STATA Corp, College Station, TX 77845, USA) to analyze all data. Missing data was ignored. Through univariate logistic regression analysis, we identified potential risk factors associated to the dependent variable using *p <* 0.10. The identified risk factors were then included in multivariate logistic regression analysis.

To reduce collinearity in multivariate analysis, we included the risk factor with the highest odds ratio (OR) in univariate analysis from factors that described the same aspect, e.g., BMI, height and BSA. If in doubt we ran the model with the competing variables and chose that which yielded the highest performing multivariate model. Thus, the following variables were excluded from multivariable analysis: age, sex, height, weight, BMI, BSA, creatinine levels, aortic length, aortic height, aortic tortuosity, distal abdominal aortic diameter, left subclavian artery diameter, anterior-posterior infrarenal abdominal aortic diameter and its derived calculated variables.

ACAS-AA can influence blood pressure, hemoglobin—and thrombocyte levels. Bleeding or dissection extending to the aortic valve can lower blood pressure. Bleeding can also influence hemoglobin and thrombocyte levels. These variables were therefore excluded from multivariate analysis. Considering the expected high abundance of smokers in the control group, history of smoking was also excluded.

In already published results from a parallel study on this population, our results have shown that diameters increase significantly from the sinotubular junction to the proximal abdominal aorta, as well as in the brachiocephalic trunk, after acute dissection [6]. Therefore, we excluded the following CT-derived variables from multivariate regression analysis: sinutubular junction aortic diameter, tubular ascending aortic diameter and its derived calculated variables, distal ascending aortic diameter, aortic arch diameter, proximal descending aortic diameter, descending aorta diameter, and proximal abdominal aortic diameter. However, to test the influence of including tubular ascending aortic diameter to the multivariate model, we made an additional model where it was included.

Independent risk factors in multivariate analysis were defined by p < 0.05. The diagnostic accuracy of the models were evaluated using receiver operating characteristic (ROC) curve analysis including area under the curve (AUC). Using STATA generated individual sensitivity and specificity, we calculated Youden index. The highest calculated Youden index was then used to determine the optimal sensitivity and specificity for the models.

### Ethical permission

The project received approval from the Department of Patient Safety at the National Board of Health (3-3013-2124/1), and the data protection agency. It was reported to our department’s internal database, in compliance with The Danish Code of Conduct. Data was stored in a RedCap database. Scientific ethical approval and informed consent were not required by the ethics committee as no new patient contact or intervention took place.

## Results

**Fig 2:**
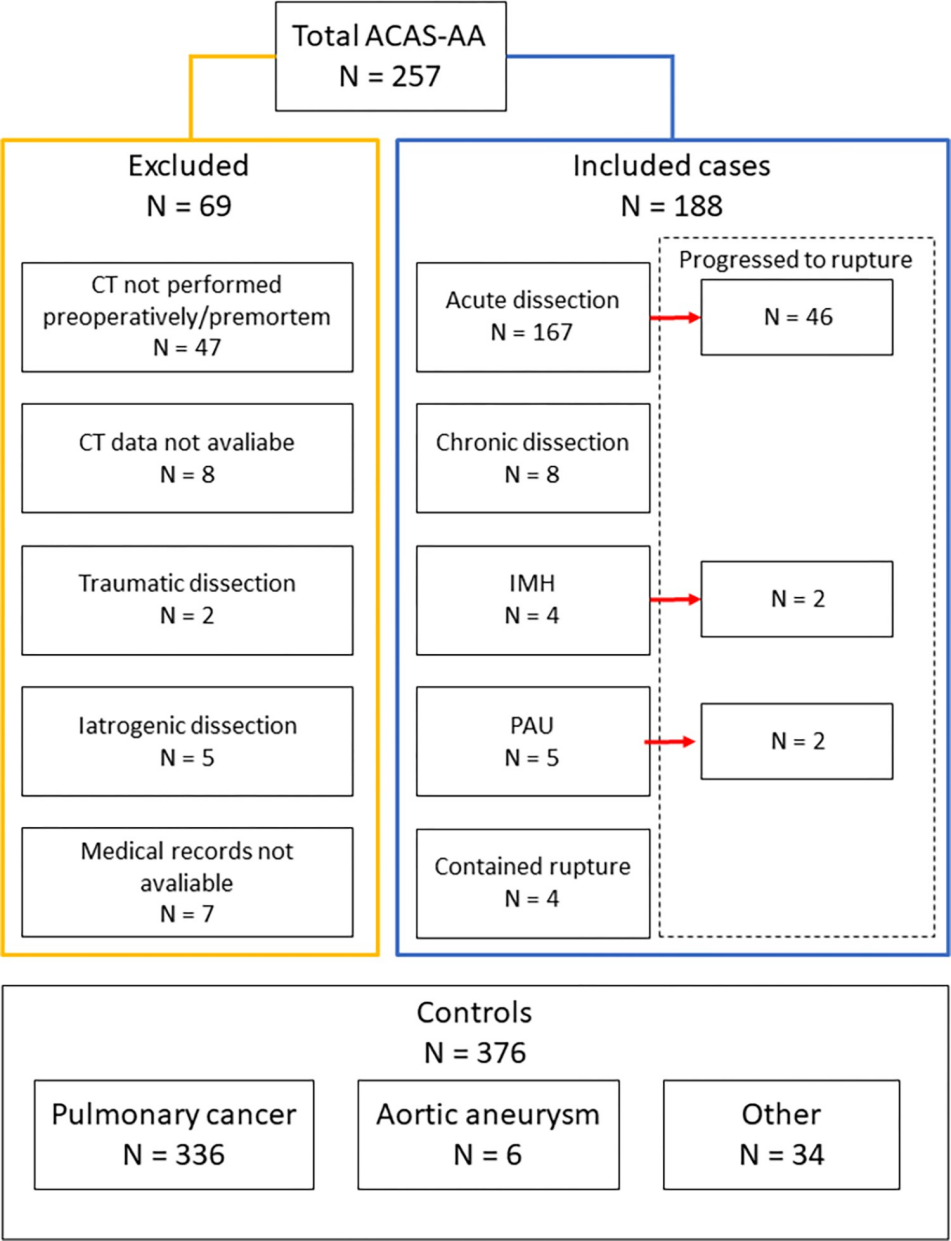
Inclusion and exclusion of cases and controls. n = number, ASAC-AA = Acute and chronic aortic syndromes of the ascending aorta, CT = computer-tomography scan, IMH = intramural hematoma, PAU = penetrating aortic ulcer. Other = patients with recurrent spontaneous pneumothorax or benign pulmonary tumors.

We included 188 cases, and excluded 69 out of a total of 257 reported ACAS-AA cases. Of the excluded ACAS-AA patients, 47 did not undergo CT-scanning prior to operation or death, 8 did not have available CT data, 2 suffered from traumatic ACAS-AA, 5 suffered from iatrogenic ACAS-AA, and 7 had no available medical records. Consequently, 376 controls were included. The control group included 336 patients diagnosed with pulmonary cancer, 6 with ascending aortic aneurysms, and 34 with benign pulmonary tumors or recurrent spontaneous pneumothorax, as detailed in Fig 2. Tables 1 and 2 summarize all variables, descriptive statistics, and results of univariate regression analysis.

The following variables were included in multivariate analysis: BAV; aortic width; renal insufficiency; history of aortic valve surgery;—hypertension,—cerebrovascular disease,—autoimmune disease and abdominal/descending aortic dissection or rupture; use of anticoagulants, use of platelet inhibitors; medio-lateral abdominal aortic diameter; LCCA diameter, diameter of first thoracic corporal body; calculated estimate of ascending aortic diameter.

Identified independent risk markers by multivariate analysis were (model one): BAV (OR 20.41, 95% CI 2.14–194.44, *p*<0.01), renal insufficiency (OR 2.9, 95% CI 1.53–5.47, *p*<0.01), medio-lateral infrarenal abdominal aortic diameter (OR 1.08, 95% CI 1.03–1.13, *p*<0.01), LCCA diameter (OR 1.4, 95% CI 1.21–1.63, *p*<0.01), and aortic width (OR 1.07, 95% CI 1.05–1.09, *p*<0.01).

The following variables were significantly associated with the independent variable when tubular ascending aortic diameter was included (model two): autoimmune disease (OR 6.86, 95% CI 1.62–5.05, *p*<0.01), medio-lateral infrarenal abdominal aortic diameter (OR 1.10, 95% CI 1.03–1.17, *p*<0.01) and tubular ascending aortic diameter (OR 1.66, 95% CI 1.47–1.87, *p*<0.01). Table 3 demonstrates the results of both models.

**Table 3:** Result of multivariate logistic regression analysis model were the presence of acute and chronic aortic syndromes of the ascending aorta is the dependent variable. Renal insufficiency was defined as eGFR < 60. Aortic width is the direct distance from the sinutubular junction to the corresponding point of the descending aorta in the transverse plane. OR = Odds ratio, CI = confidence interval.

	Model one		Model two	
Independent variable	OR (CI)	*P*-value	OR (CI)	*P*-value
**History of**				
**Abdominal/descending aortic dissection or rupture**	1.01 (0.14–7.18)	0.98	4.41 (0.27–71.658)	0.49
**Bicuspid aortic valve**	20.41 (2.14–194.44)	**< 0.01**	0.69 (0.03–14.19)	0.81
**Replaced aortic valve**	3.5 (0.74–16.38)	0.11	16.26 (0.98–268.99)	0.051
**Hypertension**	0.57 (0.31–1.06)	0.07	0.54 (0.20–1.49)	0.24
**Cerebrovascular disease**	1.69 (0.67–4.24)	0.26	2.81 (0.60–13.06)	0.18
**Renal insufficiency**	2.9 (1.53–5.47)	**< 0.01**	3.01 (0.99–7.77)	0.051
**Autoimmune disease**	2.09 (0.73–5.93)	0.16	6.86 (1.62–5.05)	**< 0.01**
**Use of**				
**Warfarin/NOAC**	1.49 (0.59–3.79)	0.39	0.92 (0.17–5.04)	0.93
**Platelet inhibitors**	0.86 (0.42–1.78)	0.69	0.67 (0.19–2.34)	0.53
**Infrarenal medio-lateral abdominal aortic diameter**	1.08 (1.03–1.13)	**< 0.01**	1.10 (1.03–1.17)	**< 0.01**
**Left common carotid artery diameter**	1.40 (1.21–1.63)	**< 0.01**	1.02 (0.86–1.39)	0.42
**Aortic width**	1.07 (1.05–1.09)	**< 0.01**	0.94 (0.94–1.02)	0.45
**First Thoracic Vertebral Corporal diameter**	0.88 (0.76–1.02)	0.10	0.87 (0.67–1.12)	0.28
**Estimated ascending aortic diameter**	0.99 (0.78–1.25)	0.93	0.78 (0.52–1.18)	0.24
**Tubular ascending aortic diameter**	-	-	1.66 (1.47–1.87)	**< 0.01**

**Fig 3:**
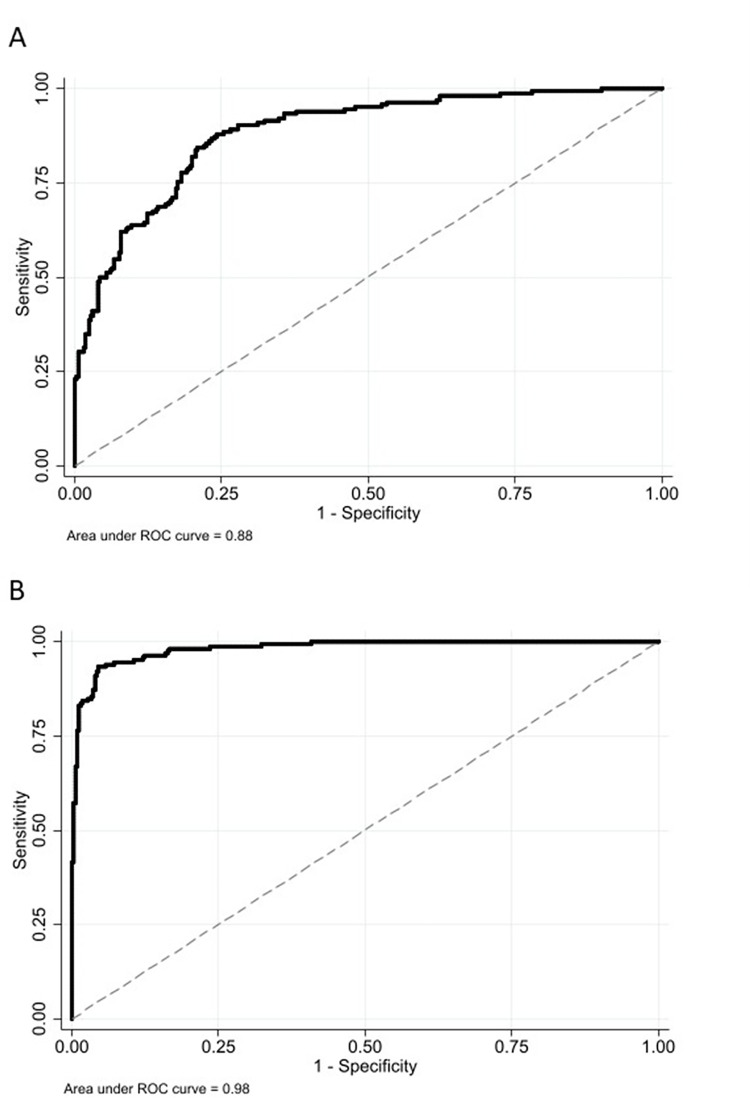
Receiver operating characteristic curve analysis of multivariate logistic regression models, where presence of acute and chronic aortic syndromes of the ascending aorta is the dependent variable. The independent variables in “A” are BAV, aortic width, renal insufficiency, use of anticoagulants, use of platelet inhibitors, medio-lateral abdominal aortic diameter, left common carotid artery diameter, diameter of first thoracic corporal body, calculated estimate of ascending aortic diameter, and history of aortic valve surgery, hypertension, cerebrovascular disease, autoimmune disease and abdominal/descending aortic dissection or rupture. The independent variables in “B” are the same as A with the addition of tubular ascending aortic diameter.

ROC curve analysis of model one yielded an estimated AUC of 0.88 (*p*<0.01), for which highest calculated Youden index was 0.63, yielding an optimal sensitivity and specificity of 87% and 75%, respectively. For model two the estimated AUC was 0.98 (*p*<0.01), the highest calculated Youden index was 0.88, with optimal sensitivity and specificity of 93% and 95%, respectively. ROC curve analysis for both models are illustrated in Fig 3.

Considering the results published by McClure et al. [16], the cumulated life risk of ACAS-AA in a Danish population aged 40 years or more, is approximately 0.2%. Using our models, positive predictive value would equal 0.6% and 3.59% in model one and two, respectively. Consequently, to prevent one event of ACAS-AA, 166 elective repairs are needed using model one, and 27 using model two.

## Discussion

In this case-control study, we explored the differences between patients suffering from acute or chronic syndromes of the ascending aorta, and a control group. Variables explored were derived from medical history and CT-scans, and tested with univariate logistic regression analysis. The association of potential risk factors was then confirmed with multivariate regression analysis. Finally, we developed two predictive models using multivariate logistic regression analysis. Model one excludes ascending aortic diameter, and model two does not.

In consistency with the literature [3, 17–20], results from the univariate analysis shows that patients suffering from ACAS-AA were more likely to be older, males, with higher BSA and frequency of cardiovascular disease,—hypertension, and use of warfarin and platelet inhibitors. Morphologically, ACAS-AA patients had larger aortic and supra-aortic vessel diameters, and longer, wider thoracic aortas.

Furthermore, our data shows that ACAS-AA patients suffer more frequently from renal insufficiency than controls. Results from population-based studies have estimated the prevalence of chronic kidney disease in patients suffering from aortic dissections to range from 8.5%– 20.4% [21]. Cases in this project had a relatively higher prevalence of renal insufficiency (37.8%), but this result might be different if the excluded cases are considered.

Identified independent risk factors for ACAS-AA in multivariate analysis model one were BAV, renal insufficiency, abdominal aortic ectasia, LCCA diameter and aortic width. The addition of tubular ascending aortic diameter to the multivariate analysis, in model two, negated the association of morphologically derived variables except abdominal aortic diameter. The association of renal insufficiency ceased, although the OR confidence interval remained largely positive. Lastly, autoimmune disease was an independent risk factor in model two, but not in model one.

The presence of BAV is a well-established risk factor for ACAS-AA [22, 23]. The task force for the diagnosis and treatment of aortic diseases of the European Society of Cardiology recommends prophylactic surgical intervention in patients with BAV and ascending aortic aneurysms equal to or exceeding 50 mm [22]. While the American College of Cardiology/American Heart Association task force guideline for the management of patients with valvular heart disease recommends surgical interventions if ascending aortic aneurysms equal to or exceeds 55 mm [23].

Chronic kidney disease is an established risk factor for atherosclerotic cardiovascular diseases [24]. Atherosclerotic lesions in the aorta can provide the bases for ulceration and subsequent intramural hematoma and rupture [22]. However, only four cases were diagnosed with intramural hematoma and five with penetrating aortic ulcer in this project. Interestingly, a recent study has found that lipid metabolism index, inflammatory factor index and M1 macrophage content, were significantly higher in patients diagnosed with dissection and atherosclerotic disease compared to patients with atherosclerotic disease [25]. These findings could suggest a link between progressing atherosclerotic disease, aortic wall degradation and increased risk of ACAS-AA.

Chronic kidney disease patients have similar characteristics to patients who suffer from ACAS-AA, such as increasing age, male gender, hypertension and higher frequency of cardiovascular diseases [24]. Nonetheless, these factors were accounted for in multivariate analysis, directly or indirectly through calculated estimated ascending aortic diameter. To the best of our knowledge, a direct association between renal insufficiency and ACAS-AA has not been reported previously.

The association of infrarenal abdominal aortic ectasia, LCCA and increased aortic width could point to a generalized pathology of the great vessels rather than one localized to the ascending aorta. Underlying systemic–and genetic diseases, as well as family history of ascending aortic disease are already established risk factors for ACAS-AA [3] and may contribute to the systemic ectasia of the great vessels.

Local inflammatory diseases, such as Takayasu arteritis or giant cell arteritis, are known risk factor for development of thoracic aortic aneurysms and ACAS-AA [3, 26]. The increased risk has also been described for systemic inflammatory diseases, such as Sjögrens syndrome or systemic lupus erythematosus [27, 28]. Thus, there seems to be some evidence suggesting that systemic inflammatory processes can induce local conditions in the aortic wall that favor ectasia and ACAS-AA.

We did not find an association between bovine aortic arch and ACAS-AA, the presence of which has consistently been shown to be associated with ascending aortic aneurysms [29–31], however the reported association to ACAS-AA has been heterogenic [10, 30, 32–35]. Similarly, we did not find an association of isolated vertebral artery to ACAS-AA. To the best of our knowledge this has yet to be investigated in other studies.

Numerous variables have been reported as direct or indirect risk factors of ACAS-AA and thoracic aortic aneurysms [3, 17, 36, 37]. These include aortitis, polycystic kidney disease, pheochromocytoma, right subclavian artery, right aortic arch, coarctation of the aorta, genetic predisposing diseases, pregnancy, use of illicit drugs, family history of aortic disease and chronic corticosteroid. Our data did not show an association of these with ACAS-AA. Some of these variables are rare in the setting of ACAS-AA. In larger settings, these risk factors might yield significant associations with ACAS-AA.

Due to data protection rights, the control group could only consist of patients treated at our respective departments. To be included, a control must have undergone CT-scanning of the thorax and preferably the abdomen. Due to the lack of screening programs for aortic ectasia, we could not include healthy controls. Therefore, the control group consisted largely of patients suffering from pulmonary cancer. These patients suffer more frequently from chronic obstructive pulmonary disease and have a history of smoking. This could explain the seemingly protective association of smoking in univariate analysis. Interestingly, in recently published DANCAVAS studies by Obel et al., smoking was associated as a protective marker for development of ascending aortic aneurysms [15, 38]. A direct protective association to ACAS-AA is however yet to be reported.

Aortic dimensions might differ in pulmonary cancer patients compared to healthy controls. Recently, an association between increasing severity of COPD and emphysema to thoracic aortic ectasia, has been reported [39]. However, the mean tubular ascending aortic diameter in the control group was similar to the reported 30–34 mm diameter in healthy subjects [40, 41].

The strength of model one is its’ reliance on surrogate markers of aortic ectasia that do not increase in diameter following dissection [6]. However, even with an AUC just shy of 90%, the model is not sufficient in selecting eligible patients from the background population for preventive repair, due to the low prevalence of ACAS-AA.

Model two is more accurate, with AUC close to 100%, and numbers needed to treat far lower than that of model one. However, the accuracy of such a model is questionable as post-ACAS-AA diameters have been shown to over-estimate pre-ACAS-AA conditions [4–7].

### Limitations

Although all cases of ACAS-AA in the region of Southern Denmark are transferred to the mentioned departments at Odense University Hospital, selection bias may have occurred. Incorrect diagnosis, death before diagnosis or death before CT-scanning has probably excluded some patients from the study. We attempted to limit further selection bias by standardizing the inclusion procedure to manuals approved by all authors. Validation of data was not possible due to the project’s retrospective setting, and some variables may be erroneously underreported. Furthermore, a control group composed of healthy individuals would have provided more accurate results.

CT-derived variables were gathered by a single investigator to ensure uniform measurement, and limit information bias. We attempted to limit confounding factors by including all described risk factors and many potential risk factors. However, not all variables were included in multivariable analysis as discussed, and therefore some confounding factors may be unaccounted for. There may also have been unidentified risk factors not included in the model. Therefore, the risk of residual confounding can´t be excluded.

## Conclusion

ACAS-AA is a complex syndrome, with both local and systemic risk factors, and predictive models for ACAS-AA should reflect this complexity. We have shown that a high performing predictive model, free of ascending aortic diameter, can be achieved. Furthermore, we have identified abdominal aortic ectasia as an independent risk factor of ACAS-AA. Integration of potential biomarkers and morphologic variables, derived from undissected aortas, would probably improve such a model.

## Abbreviations

ACAS-AA: acute and chronic aortic syndromes of the ascending aorta
BAV: bicuspid aortic valve
CT: computer tomography
BSA: body surface area
OR: odds ratio
ROC: receiver operating characteristic
AUC: Area under the curve
